# *Periplaneta americana* Ameliorates Dextran Sulfate Sodium-Induced Ulcerative Colitis in Rats by Keap1/Nrf-2 Activation, Intestinal Barrier Function, and Gut Microbiota Regulation

**DOI:** 10.3389/fphar.2018.00944

**Published:** 2018-08-22

**Authors:** Xuewei Ma, Yichen Hu, Xin Li, Xiaoting Zheng, Yitao Wang, Jinming Zhang, Chaomei Fu, Funeng Geng

**Affiliations:** ^1^School of Pharmacy, Chengdu University of Traditional Chinese Medicine, Chengdu, China; ^2^College of Pharmacy and Biological Engineering, Chengdu University, Chengdu, China; ^3^International Association of Quality Research in Chinese Medicine, Macau, China; ^4^State Key Laboratory of Quality Research in Chinese Medicine, Institute of Chinese Medical Sciences, University of Macau, Macau, China; ^5^Sichuan Key Laboratory of Medical American Cockroach, Chengdu, China

**Keywords:** ulcerative colitis, *Periplaneta americana*, 16S rRNA, gut microbiota, intestinal immunity

## Abstract

*Periplaneta americana*, a magic medicinal insect being present for over 300 million years, exhibits desirable therapeutic outcome for gastrointestinal ulcer treatment. Nowadays, *P. americana* ethanol extract (PAE) has been shown to ameliorate ulcerative colitis (UC) by either single-use or in combination with other therapeutic agents in clinics. However, its underlying mechanisms are still seldom known. Herein, we investigated the anti-UC activity of PAE by alleviating intestinal inflammation and regulating the disturbed gut microbiota structure in dextran sulfate sodium (DSS)-induced UC rats. Based on multiple constitute analyses by HPLC for quality control, PAE was administrated to DSS-induced UC rats by oral gavage for 2 weeks. The anti-UC effect of PAE was evaluated by inflammatory cytokine production, immunohistochemical staining, and gut microbiota analysis via 16S rRNA sequencing. As a result, PAE remarkably attenuated DSS-induced UC in rats. The colonic inflammatory responses manifested as decreased colonic atrophy, intestinal histopathology scores and inflammatory cytokines. In addition, PAE improved the intestinal barrier function via activating Keap1/Nrf-2 pathway and promoting the expressions of tight junction proteins. It was observed that the UC rats showed symptoms of gut microbial disturbance, i.e., the increased *Firmicutes/Bacteroidetes* ratio and the significantly decreased probiotics such as *Lactobacillus*, *Roseburia*, and *Pectobacterium*, which were negatively correlated with these detected pro-inflammatory cytokines (secreted by immune CD^4+^ T cells, and including IFN-γ, TNF-α, IL-6, IL-8, IL-17, IL-1β). Besides, PAE administration regulated the abnormal intestinal microbial composition and made it similar to that in normal rats. Therefore, PAE could attenuate the DSS-induced UC in rats, by means of ameliorating intestinal inflammation, improving intestinal barrier function, and regulating the disturbed gut microbiota, especially improving beneficial intestinal flora growth, modulating the flora structure, and restoring the intestinal-immune system.

## Introduction

Ulcerative colitis (UC) is a representative chronic inflammatory bowel disease (IBD) with high morbidity worldwide, characterized by recurrent remission and relapse (Danese and Fiocchi, 2011). It is reported that the incidence of UC ranges from 35 to 100 per 100,000 people. Moreover, a global epidemiology study has reported that compared to United States and United Kingdom, Southern Europe and Asia have a more rapid growth rate of UC incidence (Hanauer, 2006; Molodecky et al., 2012). Typical symptoms include frequent bowel movement, bloody diarrhea, abdominal pain, urgency, tenesmus, and some common complications such as proctitis, rectal abscess, enterobrosis, and colon cancer (Feuerstein and Cheifetz, 2014). However, despite the increased global attention, the etiology and pathogenesis of UC remain unclear, making it one of the most challenging gastrointestinal diseases. Until now, 5-aminosalicylic acids and corticosteroids, which could regulate the imbalance between pro-inflammatory cytokines and anti-inflammatory cytokines, have been known as the important agents for UC treatment (Huang et al., 2017b). However, some potential side-effects of these drugs, such as anti-antibody reaction, allergy, infection and mutagenesis, could be brought by long-term use and therefore compromise their clinical applications (Renna et al., 2014; Gu et al., 2017). Thus, to investigate the effective anti-UC drugs with higher drug safety is of great significance and urgency.

Complementary and alternative medicines show the potentials of being effective, low-cost treatment approaches with high safety. Some plant-derived compounds such as artemisinin, taxol, camptothecin are the representative examples of natural’s gifts to medicine at present. Epidemiological surveys suggest that, the majority of patients with IBD receive traditional medicines (Kong et al., 2005; Guo et al., 2017). Some natural products such as berberine (Habtemariam, 2016), sophocarpidine (Wang et al., 2012), indigo naturalis (Wang Y. et al., 2017), andrographolide (Liu et al., 2014), and curcumin (Sreedhar et al., 2016) also exhibit efficacy in treating UC. In addition, the medicinal insects, whose species are more than twice as many as medical plants, have also received extensive research interests. *Periplaneta americana*, more widely known as American cockroach, was first recorded in an ancient Chinese pharmacopeia “*Shen Nong Ben Cao Jing*.” As one of the largest and oldest insect groups being present for over 300 million years, this miraculous insect has also been employed as a traditional Chinese medicine for over 2,000 years, for its functions of activating blood circulation, dissipating blood stasis, promoting digestion, and inducing diuresis. Modern studies have revealed its various bioactivities (Srivastava et al., 2011; Zhang et al., 2013; Huang et al., 2017a; Shen et al., 2017; Zhao et al., 2017) such as gastric protection, wound healing, antitumor activity, immune enhancement, liver protection, and antiviral effect. *P. americana* has been developed into clinical drugs under the approval of China Food and Drug Administration (CFDA), such as “*Kangfuxin* oral liquid (Z51021834),” “*Xinmainong* injection (Z20060443),” and “*Ganlong* capsule (Z20050113).” Particularly, *Kangfuxin* oral liquid, which is prepared by the ethanol extract of *P. americana*, has been widely demonstrated for its wound healing effect in peptic ulcer treatment (Chen et al., 2016; Shen et al., 2017). In view of its significant role in gastric protection, Li et al. (2016) has been demonstrated for the therapeutic effects of *P. americana* in UC rats induced by dinitrochlorobenzene and acetic acid, which are attributed to the anti-inflammation function and fibroblasts viability. Previous studies (He et al., 2012; Zhang H. C. et al., 2017) have also indicated that, the anti-inflammation activity in UC model are related to the regulation of inflammation cytokines including iNOS, MPO, IL-4, IL-10, EGF, MUC2, and IL-6. Moreover, the mucositis amelioration of *Kangfuxin* liquid was also observed in Randomized Phase III Clinical Study (Luo et al., 2016). However, to date, the chemical constitutes and the underlying anti-UC mechanisms of *P. americana* have not been well described.

Several signal transduction cascades and transcription factors are involved in the pathological inflammatory process, such as NF-κB, MAPK, JAK-STAT, and Keap1/Nrf2/ARE (Ahmed et al., 2017) signaling pathways. At present, an increasing number of researches (Yang et al., 2016; Ahmed et al., 2017) have begun to focus on the role of Nrf2/Keap1 pathway in the inflammation-associated pathogenesis, as well as the suppression of pro-inflammatory signaling pathways. Besides, previous studies have demonstrated the decreased expression of Nrf2 in dextran sulfate sodium (DSS)-induced UC model and the important role of Nrf2 signaling pathway in UC treatment by some chemo-preventive agents (Dae Park et al., 2017; Seo et al., 2017; Liu et al., 2018; Tan and Zheng, 2018).

Up to now, although the development of IBD has been confirmed to be related to various factors including immune dysregulation, genetic disorder and barrier dysfunction, the pathogenesis of IBD still remains unclear. Gut microflora has been recorded as the highest cell density in any ecosystem, with a profound and crucial influence on human health. And the close relationship between gut microflora dysfunction and IBD has been widely acknowledged (Ohkusa and Koido, 2015). According to relevant reports, fecal bacteria from healthy donors are expected to have therapeutic effects on patients with IBD (Anderson et al., 2012). In particular, gut microflora is crucial for the protection of intestinal mucosa, and microbiota dysbiosis could lead to mucosal injury. Studies (Tao et al., 2017; Wang K. et al., 2017; Wang Y. et al., 2017) have indicated that the coexistence between the host and gut microbiota is beneficial for modulating the intestinal mucosal immune system. Gut microbiota is considered to be relevant to intestinal local inflammation and mucosal immune system. It is well known that antimicrobial drugs could generally improve IBD by modulating the host microbiota (McIlroy et al., 2017). The anti-inflammation activity of *P. americana* on gastric protection has been reported in previous studies. However, whether the anti-UC effect of *P. americana* is involved in its gut microbiota regulation activity still remains unclear. In this study, we evaluated the anti-UC activity of ethanol extracts of *P. americana* (PAE), which were provided by GoodDoctor Pharmaceutical Co., Ltd., and widely used as the preparation materials for *Kangfuxin* liquid, in a dextran sodium sulfate-induced UC rat model that mimicked many histopathological and immune characteristics of human intestinal inflammation. Meanwhile, the PAEs were characterized by means of high performance liquid chromatography (HPLC) quantification for quality control. Eventually, the anti-UC effects and mechanisms of the PAEs related to anti-inflammation, intestinal barrier improvement, and gut microbiota regulation were evaluated.

## Materials and Methods

### Chemicals and Materials

*P. americana* ethanol extracts (PAE) were extracted from *P. americana* provided by Good Agricultural Practice (GAP) breeding base of GoodDoctor Pharmaceutical Co., Ltd. (Sichuan, China), while *P. americana* water extracts were obtained by water extraction following the steps below: after being degreased with petroleum ether, *P. americana* crude powders (100 g), obtained from the Good Agricultural Practice (GAP) breeding base of GoodDoctor Pharmaceutical Co., Ltd. (Sichuan, China), was soaked for 2 h, extracted with water for 10 times, boiled for 30 min, and finally collected by freeze-drying after filtration. Both ethanol and water extracts of *P. americana* were dissolved in DMSO for cell experiments and then suspended in 0.5% CMC-Na solution for animal test, respectively. HPLC-grade acetonitrile and water used in this study were purchased from Fisher Scientific UK.

### HPLC Analysis

Chromatographic analysis was performed on an Agilent 1260 series HPLC system equipped with a diode array detector (DAD), using a Zorbax XDB C_18_ (4.6 mm × 250 mm, 5 μm) at a column temperature of 25°C. The flow rate and injection volume were 1 ml/min and 10 μl, respectively. The methanol–water (2.5:97.5) system was employed as the mobile phase for quantitative determination of multiple standard compounds, i.e., guanosine, uridine, inoside, cytidine, hypoxanthine, thymine, adenine, cytosine, and uracil. All these standard compounds were dissolved together by 3% methanol to form a mixed standard solution. The optimized detection wavelength was 254 nm.

### Anti-inflammatory Effect of PAE *in vitro*

RAW 264.7 macrophage cell line, obtained from American Type Culture Collection (ATCC; Manassas, VA, United States), was cultured in RPMI-1640 medium supplemented with 10% FBS, 100 U/ml penicillin and 100 μg/ml streptomycin. All cells were incubated in a humidified 5% CO_2_ incubator at 37°C, and plated in 96-well plates at densities of 1 × 10^4^ cells/well. The concentrations of non-toxic PAE in RAW 264.7 were evaluated by MTT assay described in supplementary materials in the first place, and the effects of PAE on the pro-inflammatory cytokine production in RAW 264.7 cells were then explored. After 24 h adherence, the cells were pre-incubated in a FBS-free medium containing a series of amounts of PAE for 2 h, and then treated with 1 μg/ml of LPS stimulation for 12 h. After then, cell culture supernatants were collected and assayed for NO production by reacting with Griess reagent (Hu et al., 2017) and the amounts of inflammatory cytokines, including TNF-α, IL-1β, PGE_2_, were calculated by an enzyme-linked immunosorbent assay (ELISA) kit for rats (Ge et al., 2017) (R&D Systems, Inc., Minneapolis, MN, United States). Meanwhile, the cell viability was assessed by incubating with MTT regent for 4 h, and the absorbance of DMSO solution containing formazan crystal was measured at 570 nm (Zhang J. et al., 2017).

### Anti-UC Effects in DSS-Induced UC Rats

In view of the high reproducibility in experimental implementation and the high similarity with the features of clinical UC, the DSS-induced UC model is widely recognized and frequently used to evaluate the anti-UC effects involved in the anti-inflammatory approaches, in which cases, repeated administration of DSS could result in the disruption of the colonic mucosal architecture and long-term chronic inflammation. Herein, the UC rat model was established by oral administration of 5% DSS (w/v) dissolved in drinking water for seven consecutive days. The UC rats were randomly divided into four groups (*n* = 8) with various treatments for 14 consecutive days, i.e., the model group, the high-dose PAE group, the low-dose PAE group, and the *P. americana* water extract group (W-E). More specifically, rats in model group were given only saline by gavage administration once per day. UC rats in high- and low-dose PAE groups were administrated with 200 mg/kg and 100 mg/kg of PAE, respectively. Since water decoction is a traditional preparation approach of Chinese medicine, W-Es (200 mg/kg) of *P. americana* herein were obtained and evaluated to determine whether they could alleviate UC-model rats and serve as the counterpart of PAE. During the administration of various agents, 3% DSS (w/v) was given to rats to avoid self-cure. Apart from these UC rats, rats without DSS treatment were only given saline in the control group.

The weight loss, stool consistency and occult/gross bleeding of all rats were recorded every 3 days throughout the experiment. Disease activity index (DAI) scores were calculated based on the previously described evaluation standard (Cho et al., 2011), to assess the extent of UC. After 14 days of administration, all rats were sacrificed by isoflurane inhalation, and their colon samples were collected and exampled. Part of the colon segments were fixed by 4% paraformaldehyde, embedded in paraffin and then cut into 4 μm thick sections. These sections were stained with hematoxylin and eosin (HE) and alcian blue, respectively, in accordance with the standard procedures for histopathological analysis. Additionally, the remaining colon segments were weighted and homogenized with 0.1 M phosphate buffer (pH 7.4). The homogenate suspension was collected by centrifugation at 5,000 rpm for 15 min. The amounts of inflammation-associated cytokines including IFN-γ, IL-17, IL-8, IL-6, IL-1β, and TNF-α were determined using ELISA kits according to the manufacturers’ instructions.

### Intestinal Barrier Function Activities

The intestinal barrier functions were evaluated via Keap1 and Nrf-2 mRNA analysis by quantitative reverse transcription PCR (qRT-PCR) and the expressions of tight junction proteins (ZO-1, occludin, and claudin-1) were via Western blot analysis. Total RNA of colon tissue was extracted by TRIzol reagents according to the manufacturer’s instructions (Invitrogen, United States). The PCR reaction mixture contained 2 × qPCR mix, 7.5 μM PCR primer, 2.5 μl reverse transcription product and 8.0 μl ddH_2_O. The RT quantitative reaction was performed as follows: precycling at 95°C for 10 min, then 40 cycles of denaturation at 95°C for 15 s and annealing at 60°C for 60 s. Primers for Keap1, Nrf2, and GAPDH were listed in **Table 1**. Fluorescence was measured at the end of each annealing step, and the melting curves were monitored to identify the specificity of the PCR products. The 2^−ΔΔCt^ method was used to determine the mRNA expression levels of Keap1 and Nrf-2 relative to the control gene GAPDH.

**Table 1:** Primers for real-time quantitative PCR.

Gene	Primer sequences	Product size (bp)
Keap1-S	5′-TATGAGCCAGAGCGGGACGA-3′	172
Keap1-A	5′-TCATCCGCCACTCATTCCTCT-3′	
Nrf-2-S	5′-CTGGCTGATACTACCGCTGTTC-3′	208
Nrf-2-A	5′-AGGTGGGATTTGAGTCTAAGGAG-3′	
GAPDH-S	5′-AGGAGCGAGACCCCACTAACA-3′	247
GAPDH-A	5′-AGGGGGGCTAAGCAGTTGGT-3′	

The colon segments were homogenized with RIPA buffer containing protease inhibitor (1:100). After protein concentration was determined by BCR protein assay kit, Western blot assay was performed as described (Kang et al., 2017). The primary antibodies against ZO-1, occludin and claudin-1 were then incubated overnight at 4°C. GAPDH antibody was used as an internal control to ascertain the equal loading of proteins. The obtained chemiluminescence bands were analyzed with ImageJ software.

Furthermore, intestinal epithelial permeability *in vivo* was determined via FITC probe based on the previously described method (Moussaoui et al., 2014). Briefly, UC model rats were employed and administrated with saline, high-dose PAE, low-dose PAE, and W-E for 14 consecutive days. After then, these UC rats and normal rats were fasted overnight and given fluorescein isothiocyanate (FITC)-dextran solution (4 kDa, 600 mg/kg) by gavage. Their blood samples were collected from retinal veins 4 h after administration and then centrifuged at 3,000 × *g* and 4°C for 10 min. Serum levels of FITC were measured at 480 and 520 nm using a microplate reader.

### Effects on Gut Microbiota

In light of the anti-UC effects of high-dose PAE, whether PAE could regulate gut microbiota was investigated. Primarily, total genomic DNAs from the feces of rats in various groups were extracted using DNeasy PowerSoil Kit (QIAGEN) according to the descriptions. DNA concentration and quality were checked using a NanoDrop Spectrophotometer. Bacterial 16S rRNA gene sequences (V4 region) were amplified by PCR instrument (Applied Biosystems^®^ Gene Amp^®^ PCR System 9700) using primer 5′→3′ (Caporaso et al., 2011): 515F (5′-GTGCCAGCMGCCGCGGTAA-3′) and 806R (5′-GGACTACHVGGGTWTCTAAT-3′). The amplification procedures were conducted at 94°C for 1 min in the first place, and then followed by 30 cycles (denaturation at 94°C for 20 s, annealing at 54°C for 30 s, and elongation at 72°C for 30 s) and a final extension at 72°C for 5 min. PCR reactions were performed in triplicate: 25 μl mixture containing 1x PCR buffer, 1.5 mM MgCl_2_, each deoxynucleoside triphosphate at 0.4 μM, each primer at 1.0 μM, 0.5 U of KOD-Plus-Neo (TOYOBO) and 10 ng template DNA. Amplicons were extracted from 2% agarose gels, purified with the OMEGA Gel Extraction Kit (Omega Bio-Tek, United States) and then quantified with Qubit @ 2.0 Fluorometer (Thermo Fisher Scientific, United States).

MiSeq Illumina sequencing was further processed on the sequencing reaction (Illumina Inc., San Diego, CA, United States) for paired-end reads to establish a DNA library using the TruSeq DNA PCR-Free Sample Prep Kit (FC-121-3001/3003) as per the standard protocols. The paired-end reads were merged by FLASH and then assigned to each sample according to the unique barcodes, so as to get rid of the low-quality tags (length < 200 bp, more than two ambiguous base ‘N,’ or the average base quality score < 30). High-quality tags were clustered into operational taxonomic units (OTUs) using UPARSE (Edgar, 2013) algorithm in QIIME software based on a 97% sequence similarity, and these OTUs were further subjected to analysis using database of Greengenes by PyNAST algorithm. Alpha and Beta diversities and principal coordinate analysis (PCoA) were analyzed by QIIME, Mothur, and R software. LEfSe analysis were done using Python LEfSe package (Segata et al., 2011).

### Statistical Analysis

The experimental data were analyzed by GraphPad Prism 6.0 software and presented as mean ± SD. The values of various groups were evaluated by one-way ANOVA and difference test. *P* < 0.05, *P* < 0.01, and *P* < 0.001, calculated using SPSS software version 17.0., were considered statistically significant.

## Results

### HPLC Quantitative Analysis

**FIGURE 1:**
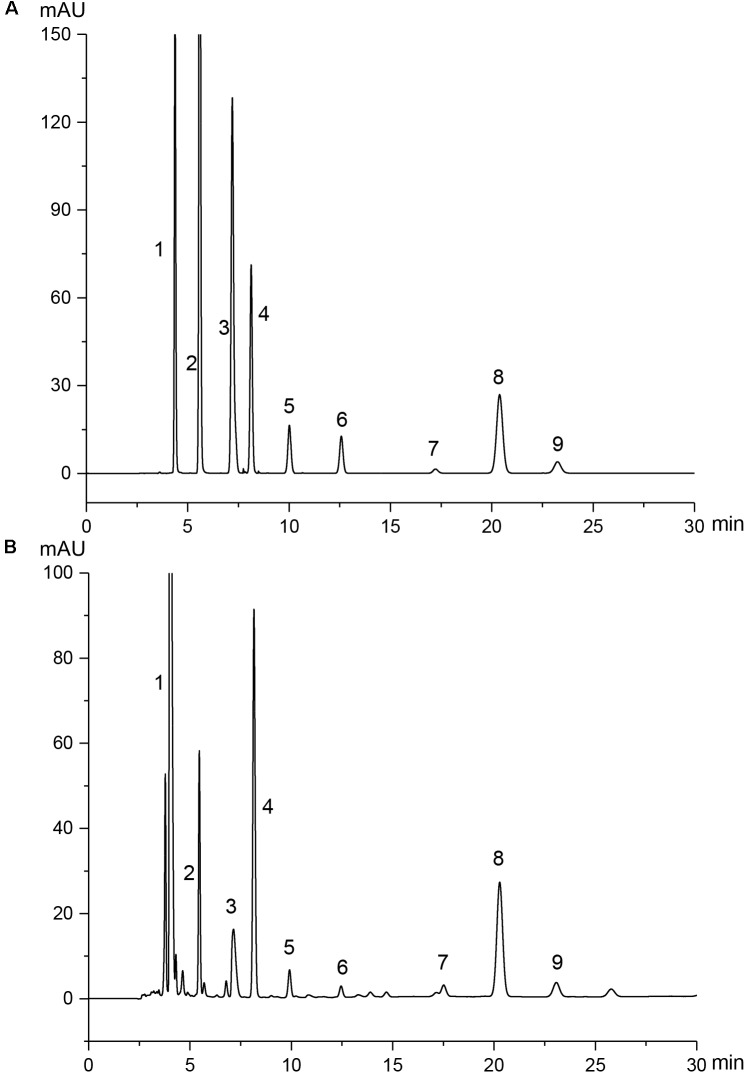
HPLC chromatograms of mixed standard substances **(A)** and PAE samples **(B)**. Peaks 1∼9 are derived from cytimidine, uracil, cytidine, hypoxanthine, uridine, thymine, adenine, inosine, and guanosine, respectively.

Given the complex composition of herbal extracts, the quantitative analysis was of great importance for the quality control of PAE herein. The amounts of nine physiological small molecules, i.e., guanosine, uridine, inosine, cytidine, hypoxanthine, thymine, adenine, cytosine, and uracil, in PAE were determined by HPLC. These nucleotides, nucleosides and nucleobases were deemed to be the potential active components in some natural products such as *Cordyceps sinensis* (Yuan et al., 2008; Yang et al., 2010) and *Sipunculus nudus* (Ge et al., 2016). As shown in **Figure 1A**, these nine compounds in mixed standard substances were analyzed, with a satisfied degree of separation and methodological investigation being obtained. The standard curves and linear ranges of these compounds were as follows: cytimidine: *y* = 132.64*x*-3.1286 (*r*^2^ = 0.9999, 0.311∼6.22 mg⋅ml^−1^), uracil: *y* = 216.56*x*+ 0.3047 (*r*^2^ = 1, 0.432∼8.64 mg⋅ml^−1^), cytidine: *y* = 79.1*x*-0.1193 (*r*^2^ = 1, 0.354∼7.08 mg⋅ml^−1^), hypoxanthine: *y* = 153.25*x*-18.512 (*r*^2^ = 0.9996, 0.392∼7.84 mg⋅ml^−1^), uridine: *y* = 22.569*x*-1.056 (*r*^2^ = 1, 0.389∼7.78 mg⋅ml^−1^), thymine: *y* = 28.056*x*+ 2.0681 (*r*^2^ = 0.9990, 0.272∼5.44 mg⋅ml^−1^), adenine: *y* = 4.1931*x*+ 3.7809 (*r*^2^ = 0.9997, 0.363∼7.26 mg⋅ml^−1^), inosine: *y* = 100.13*x*+ 40.147 (*r*^2^ = 0.9993, 0.302∼6.04 mg⋅ml^−1^), guanosine: *y* = 15.581*x* + 12.32 (*r*^2^ = 0.9992, 0.304∼6.08 mg⋅ml^−1^). **Figure 1B** provides clear evidence that the concentrations of these nine compounds in PAE sample could be determined. Accordingly, based on the external standard method, the amounts of these nine compounds, i.e., cytimidine, uracil, cytidine, hypoxanthine, uridine, thymine, adenine, inosine, and guanosine, in PAE were calculated and the results were 6.165, 0.724, 1.375, 2.466, 1.422, 0.451, 2.885, 2.373, and 1.737 mg/g, respectively. This result indicated that the multicomponent quantification method could provide a definite quality control approach for PAE.

### The Expressions of Pro-inflammatory Factors Were Down-Regulated in LPS-Stimulated RAW 264.7 Cells

**FIGURE 2:**
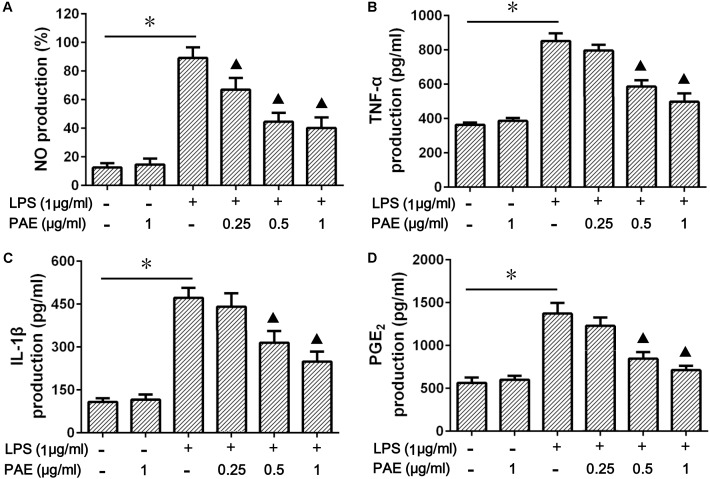
Effects of PAE on the production of NO **(A)**, TNF-α **(B)**, IL-Iβ **(C)**, and PGE_2_ **(D)** in LPS-stimulated RAW 264.7 macrophages. RAW 264.7 cells were pre-incubated in FBS-free medium containing a series of amounts of PAE for 2 h, and then co-treated with 1 μg/ml of LPS stimulation for 12 h. ^∗^*P* < 0.05 blank control group or PAE vs. LPS-stimulated group, ^
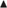
^*P* < 0.05 LPS + PAE groups vs. LPS-stimulated group (*n* = 6 per group).

Macrophages are the major source of pro-inflammatory cytokines. In order to investigate the inhibition effects of PAE on inflammatory factor expression, the amounts of NO, TNF-α, IL-Iβ, and PGE_2_ in LPS-stimulated RAW 264.7 cells were determined by Griess reaction and ELISA assay. The potential cytotoxicity of PAE on RAW 264.7 cells was first evaluated by MTT assay, indicating that PAE (0.25, 0.5, and 1 μg/ml) was not significantly cytotoxic after 24 h incubation (**Supplementary Figure S1**). As is well known, the NO amount in RAW 284.7 cell culture medium could be dramatically increased by the simulation of LPS. Whether PAE could attenuate LPS-induced NO production in RAW 264.7 macrophages was then examined. The significant reduction in NO production induced by the LPS-simulated macrophages was due to the addition of PAE in a concentration-dependent manner (**Figure 2A**). Subsequently, the changes of inflammatory factor production were evaluated based on its non-toxicity against macrophage cells. As shown in **Figures 2B–D**, compared with the blank control or PAE group, LPS induced a more significant increase in the production of pro-inflammatory cytokines (by 2.34-fold for TNF-α, 4.37-fold for IL-Iβ, and 2.44-fold for PGE_2_). However, the pretreatment of PAE with various concentrations could significantly inhibit the LPS-induced production. These results indicated that PAE could remarkably alleviate the inflammatory reaction by inhibiting the production of nitric oxide and pro-inflammatory cytokines.

### PAE Attenuated DSS-Induced UC in Rats

**FIGURE 3:**
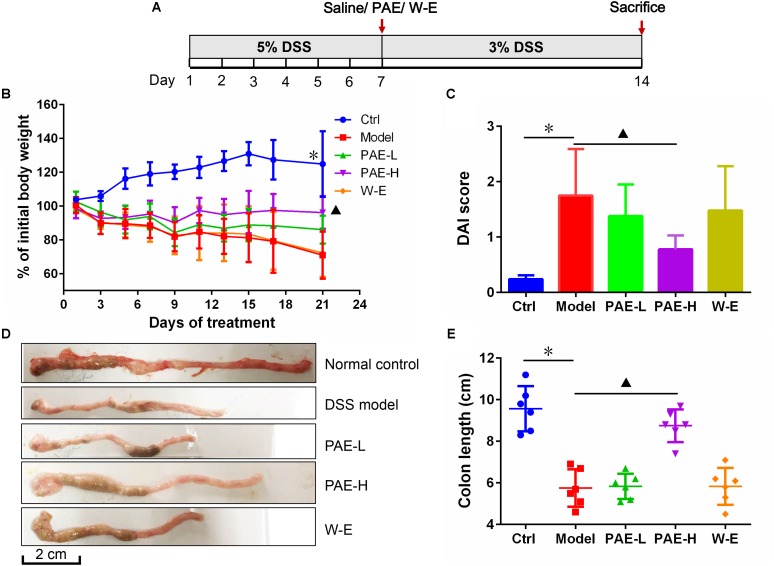
Effects of PAE treatment on the of DSS-induced UC rats after 14 days of continuous gavage. **(A)** The medication regimen. **(B)** Rats’ body weight changes from day 1 to day 21 throughout the experiment. **(C)** DAI scores of rats in various groups. **(D)** Representative photographs of rats’ colons in various groups. **(E)** Colon length of rats in various groups. ^∗^*P* < 0.05 untreated control group vs. DSS-induced UC model group, ^
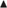
^*P* < 0.05 model group vs. PAE-H group (*n* = 6 per group).

Preliminary experiments have shown that continuous administration of 5% DSS in drinking water for more than five consecutive days could induce various symptoms, such as diarrhea and even hematochezia, which exhibit similarities with human UC. However, if the 5% DSS is replaced with fresh water, these symptoms in rats would gradually disappear after 7 days. Therefore, to avoid the self-cure of UC rats after the termination of 5% DSS administration, 3% DSS was used to keep the pathological condition during the administration of PAE agents herein (**Figure 3A**). The body weight changes of the rats throughout the experiment clearly reflected their physiological status. **Figure 3B** shows a significant body weight loss of DSS-treated UC rats at day 21, in comparison with the control group. However, rats in PAE-H group exhibited a less distinct weight loss compared with the model group. In view of these representative clinical features, the DAI was used to evaluate the therapeutic activity of PAE. As shown in **Figure 3C**, DAI scores of rats treated with DSS were significantly increased in comparison with those of the control group. However, DAI scores of PAE-H group remarkably dropped, indicating the improvement effect of high-dose PAE on UC-related pathological states. In addition to the severe synechia, hyperemia, edema, the colon length of rats treated with DSS was apparently shortened. **Figures 3D,E** show the colon shortening of rats treated with DSS. Given all that, high-dose PAE could significantly ameliorate the colon shortening.

### PAE Ameliorated the Histopathological Changes in Colon Tissues

**FIGURE 4:**
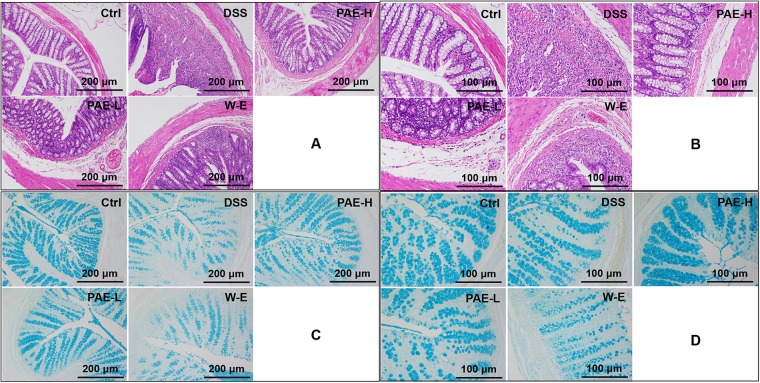
Effects of PAE on DSS-induced UC rats by HE staining and alcian blue staining of colorectum sections (**A** and **C** with magnification ×100, **B** and **D** with magnification ×200).

Based on the above-mentioned findings, DSS could induce severe inflammation of colon tissues. The histopathological screening of rats’ colon sections by HE-staining revealed no necrosis or inflammation in the control group (**Figures 4A,B**). Rats in the DSS model group showed colon tissue injuries, including crypt distortion, goblet cell loss, severe epithelia damage, and mucosal inflammatory cell infiltration (Chen et al., 2015). However, the administration of high-dose PAE could significantly protect colon crypt structure and reduce histologic inflammation. Moreover, it was hypothesized that PAE could protect the intestinal mucosa from damages by reinforcing its self-repair. To further demonstrate this hypothesis, alcian blue staining was conducted to investigate the intestinal mucosa changes after PAE administration during the DSS treatment. **Figures 4C,D** show that DSS could remarkably decrease the expressions of mucins, which were involved in the repair of colon mucosa, compared with the control group. Nevertheless, both high- and low-dose PAEs could increase the mucin expressions, showing much more positive blue cells in both PAE-H and PAE-L groups. These results suggested that PAE could inhibit colonic inflammation and promote the restoration of intestinal mucosa.

### PAE Reduced the Expressions of Inflammatory Cytokines in the Colorectum Tissues of UC Rats

**FIGURE 5:**
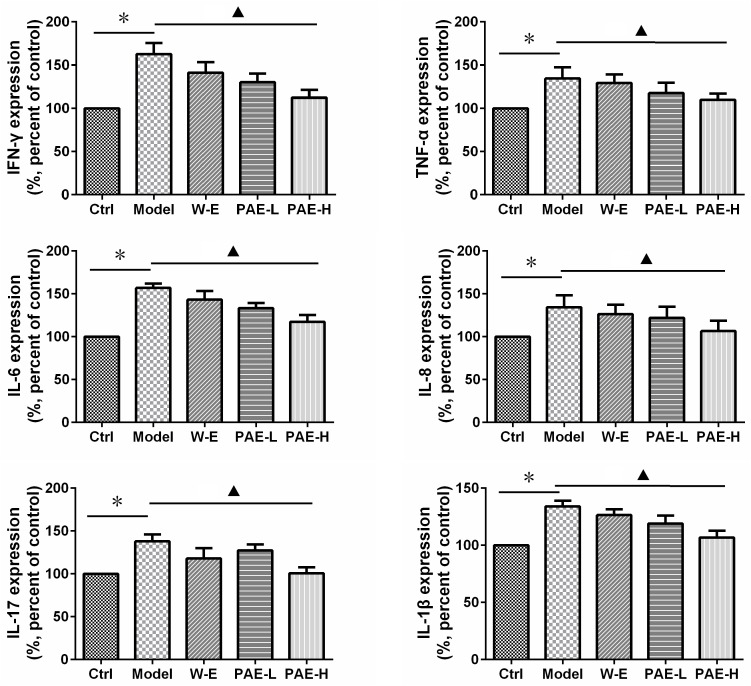
Effects of PAE on the amounts of inflammatory cytokines, including IFN-γ, TNF-α, IL-6, IL-8, IL-17, IL-1β, in the colorectum tissues of DSS-induced UC rats by ELISA measurement. ^∗^*P* < 0.05 untreated control group vs. DSS-induced UC model group, ^
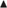
^*P* < 0.05 model group vs. PAE-H group (*n* = 6 per group).

In the intestinal mucosal immune system, neutrophils and macrophages are responsible for inflammatory cytokine secretion, which could disrupt epithelial integrity and cause colon injury (Grisham and Yamada, 1992). The production of pro-inflammatory cytokines was significantly increased in DSS-induced model group compared with the control group. However, both PAE and water extract reduced the abnormally increased amounts of pro-inflammatory cytokines (**Figure 5**). It has been widely demonstrated that the increased amounts of TNF-α, IL-6, and IL-1β were related with the inflamed mucosa and superficial ulcers in UC rats (Pavlick et al., 2002). Additionally, IFN-γ, IL-8, and IL-17 also served as the pro-inflammatory cytokines to evaluate the UC status (Qu et al., 2017). As a result, high-dose PAE significantly diminished these raised pro-inflammatory cytokines, with the most potent efficacy. These results suggested that PAE could regulate the expressions of pro-inflammatory cytokines, providing an evidence for the remission of epithelial cell necrosis, edema, and neutrophil infiltration.

### PAE Improved the Intestinal Mucosal Barrier Function by Keap1/Nrf-2 mRNA and the Expressions of Tight Junction Proteins

**FIGURE 6:**
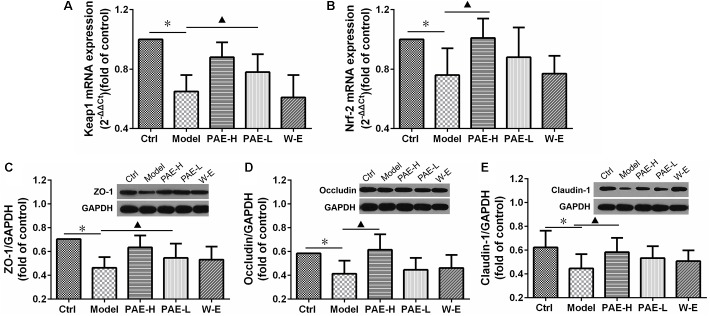
Effects of PAE on the intestinal mucosal barrier function. mRNA expressions of Keap1 **(A)** and Nrf-2 **(B)** determined by real-time quantitative PCR analysis. Expressions of ZO-1 **(C)**, occludin **(D)**, and claudin-1 **(E)** over GAPDH determined by Western blot analysis. ^∗^*P* < 0.05 untreated control group vs. DSS-induced UC model group, ^
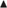
^*P* < 0.05 model group vs. PAE-treated group (*n* = 6 per group).

Substantial evidence indicates that UC is associated with oxidative stress, which may play a significant role in its etiologies (Patlevic et al., 2016). It has been substantially demonstrated that nuclear factor (erythroid derived 2)-like 2 (Nrf2), a redox-sensitive transcription factor, as well as its tight interact with Kelch-like ECH-associated protein 1 (Keap1) could promote the antioxidant activity and provide therapeutic benefits in inflammation and associated disorders. Keap1/Nrf-2 signaling pathway plays a key role in the protection of intestinal cells against oxidative stress (Yang N. et al., 2017). Therefore, the mRNA expressions of Keap1 (**Figure 6A**) and Nrf-2 (**Figure 6B**) were measured by real-time quantitative PCR. As shown in the previous figures, the Keap1 and Nrf-2 mRNA expressions of rats in the UC model group were down-regulated, compared with normal rats. However, the administration of high-dose PAE significantly increased the mRNA expressions of both Keap1 and Nrf-2, indicating that PAE could activate Keap1/Nrf-2 signaling pathway to resist the intestinal mucosal injury.

Furthermore, the protein expressions of ZO-1, occludin and claudin-1 were measured by Western blot analysis to evaluate the tight junction integrity of the colon (Zhang L. C. et al., 2017). As shown in **Figures 6C,D,E**, the amounts of all these proteins were decreased in UC rats compared with the normal rats in the control group, indicating that the tight junction integrity was compromised. However, PAE promoted the expressions of these proteins remarkably, indicating its improvement on the tight junction structure and promotion on the intestinal mucosal barrier function. Comparatively speaking, the water extract could not up-regulate the mRNA expressions of Keap1/Nrf-2 and tight junction-related proteins, which was in accord with its negative anti-UC activity as mentioned above.

### PAE Modulated Gut Microbiota in Ulcerative Colitis Rats

**FIGURE 7:**
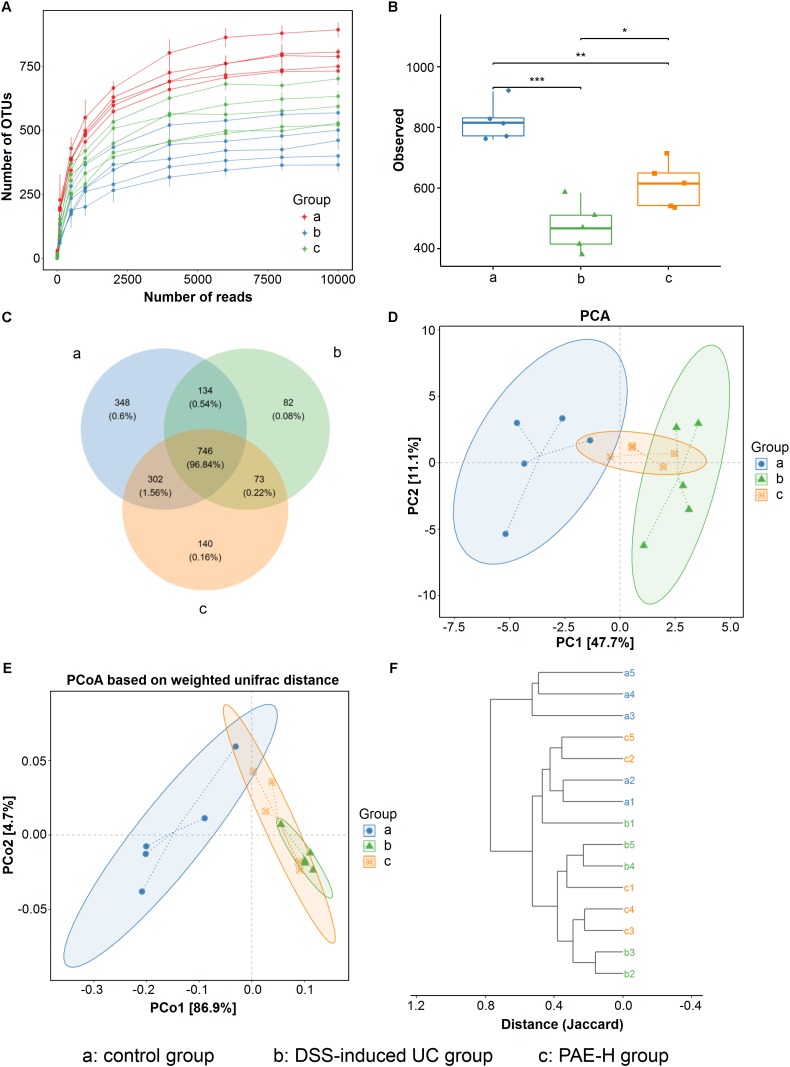
PAE regulation on the disturbed gut microbiota in DSS-induced UC rats. **(A)** Rarefaction curves determined at the 97% similarity level. **(B)** Alpha diversity analyzed by Shannon diversity index. **(C)** Venn diagram of OTUs in the three groups. **(D)** PCA analysis of variation. **(E)** PCoA analysis of variation based on the weighted UniFrac distance. **(F)** Cluster dendrogram of the three groups based on Jaccard distance. Significant difference among two group was set as ^∗^*P* < 0.05, ^∗∗^*P* < 0.01, ^∗∗∗^*P* < 0.001 (*n* = 6 per group).

The expression of 16S rRNA gene sequence was used to evaluate the amelioration of high-dose PAE on DSS-induced UC. Totally, the mean values of 10141 effective sequences in each sample were collected to generate 1,825 OTUs, based on a similarity of at least 97%. The rarefaction curve plateau (**Figure 7A**) with the current sequencing indicates that most of the diversities have already been captured in all samples. It appeared that UC model rats possessed the least number of species, compared with the normal rats and the UC rats administrated with high-dose PAE. As shown in **Figure 7B**, Shannon index was used to indicate the alpha diversity of microbial communities, showing a significant decrease of the alpha diversity due to DSS treatment and a remarkable increase of microbial diversity due to the administration of PAE-H. As shown in the Venn diagram (**Figure 7C**), 746 OTUs (96.84%) overlapped among these three groups, 34 OTUs (0.54%) between the control and model groups, 73 OTUs (0.22%) between the DSS and PAE-H groups, and 302 OTUs (1.56%) between the control and PAE-H groups. PCA (**Figure 7D**) and PCoA (**Figure 7E**) analyses based on the weighted UniFrac distance matrices suggested that the gut microbiota in three groups were significantly diversified. Although the administration of high-dose PAE could not completely reverse the gut microbiota to those of the control group, PAE still presented the function of regulating abnormal gut microbiota in DSS-induced UC rats. The system clustering tree in **Figure 7F** provides evidences of a significant difference existing among the three groups, with a higher similarity shown in the microbiota communities between the PAE-H group and the control group.

**FIGURE 8:**
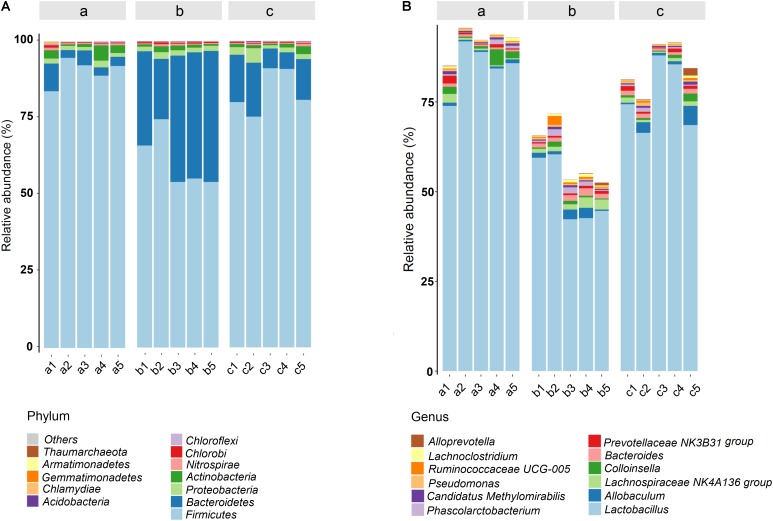
Gut microbial community structures of rats from various groups. Microbial community bar plot by phylum **(A)** and genus **(B)**. a: normal rats serve as the control group; b: UC rats induced by DSS; c: UC rats treated with high-dose PAE (*n* = 6 per group).

Histograms were used to reflect the differences among various groups on species and relative abundance of intestinal microbiota. At the phyla level (**Figure 8A**), *Firmicutes*, *Bacteroidetes*, *Proteobacteria*, and *Actinobacteria* were the predominant and most numerous species in all groups. The significantly increased *Bacteroidetes* and decreased *Firmicutes* acted as the representative characters of the DSS-induced UC rat model. The result was accordant with the rising *Firmicutes/Bacteroidetes* ratio of UC rats in a previous report (Tao et al., 2017; Yang Y. et al., 2017). However, after the administration of PAE-H, the abnormal microbiota community structure became similar to that of the untreated control group. The detailed information about the regulation effect of PAE on gut microbiota of UC rats was provided in bar plots at genus level (**Figure 8B**). As shown in the figure, 12 genera were identified in all samples. The most remarkable change on microbiota abundance in UC rats was *Lactobacillus*, in comparison with normal rats. *Lactobacillus*, a well-known probiotic (Curro et al., 2017; Amer et al., 2018), exhibited beneficial effects on inflammatory bowel disorders by stimulating immune cells, depressing pro-inflammatory cytokine secretion, and inducting anti-inflammatory cytokines. Specifically, the increase in the abundance of *Lactobacillus* was observed in PAE-H group, indicating that PAE could help to protect intestinal tract and alleviate intestinal inflammation by increasing the probiotics.

**FIGURE 9:**
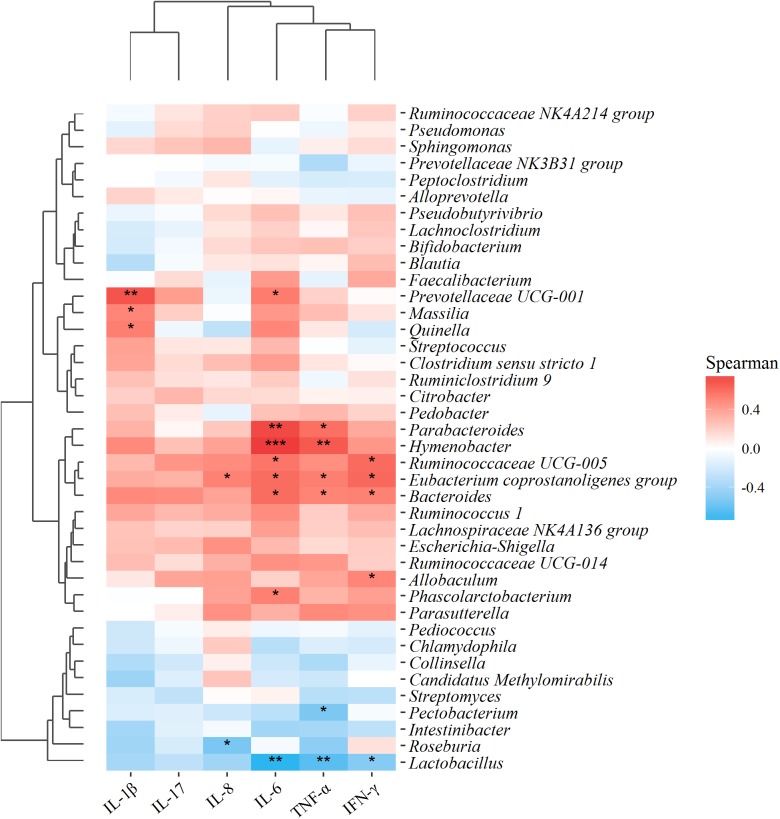
Correlation analysis between 40 microflora genera with high abundance in all samples and environmental variables, i.e., the above-mentioned six pro-inflammatory cytokines. Red and blue blocks represent the positive and negative correlations, respectively. Gradation of color indicates the correlation degree. ^∗∗∗^*P* < 0.001; ^∗∗^*P* < 0.01; ^∗^*P* < 0.05 (*n* = 6 per group).

To figure out whether the changes in gut microbial of UC rats were correlated to the intestinal inflammatory symptoms, the relationship between the 40 microflora genera with high abundance in all samples and those above-mentioned six pro-inflammatory cytokines, were analyzed using Spearman’s correlation coefficient, based on the hierarchical clustering and Heatmap. As shown in **Figure 9**, apart from IL-17, all the other pro-inflammatory cytokines exhibited significant correlations with these predominant microflora genera. Particularly, *Lactobacillus* exhibited a significantly negative correlation with inflammatory factors IL-6, TNF-α, and IFN-γ, indicating that an increase in the abundance of *Lactobacillus* would help to suppress the production of these inflammatory factors. On the contrary, the pathogenic bacteria *Bacteroides* exhibited a significantly positive correlation with IL-6, TNF-α, and IFN-γ. The results manifested that the consecutive administration of PAE could significantly alleviate the intestinal inflammation (shown in **Figure 5**), which was greatly associated with the increase of *Lactobacillus* and the down-regulation of *Bacteroides* in UC rats.

## Discussion

It is known that the pathogenic manifestations of UC include defects in epithelial barrier, immune response, leukocyte recruitment and colonic microflora, but the underlying specific etiologic causes and mechanisms still remain unknown (Lynch and Hsu, 2017). In particular, UC frequently leads to intestinal epithelium injury, resulting in the generation of endogenous and exogenous antigens and the bacteria translocation via the portal venous system. Intestinal barrier defects are common in IBD patients and believed to increase the uptake of luminal antigens across the intestinal epithelium, which in turn would trigger the immune system and the development of mucosal inflammation (Vancamelbeke et al., 2017). Given the limited therapeutic effect of the current approaches focusing on immune response, improvement of intestinal barrier function in intestinal epithelial cells may provide new avenues for UC treatment characterized by changes in barrier function. Tight junctions and adherence junctions are primarily responsible for the restriction and modulation of intestinal permeability. Samak et al. (2015) found that DSS treatment could increase tyrosine phosphorylation of occludin and ZO-1 in Caco-2 cells, indicating the disruption of tight junctions. A previous study (Lahey et al., 2017) has demonstrated the promotion of tight junction, composed of various transmembrane proteins such as occluding, claudin family proteins and intracellular zona occludens (ZO) family proteins, could help to alleviate UC symptoms. In present study, representative proteins of tight junctions, i.e., ZO-1, occludin and claudin-1, were evaluated, showing that DSS treatment could down-regulate the tight junction proteins in colons of UC rats (**Figure 5**). Additionally, the intestinal epithelial tight junction serves a key role in protecting against inflammation, and the disrupted tight junction is a main cause of intestinal barrier dysfunction and inflammation. Therefore, whether PAE could regulate intestinal permeability *in vivo* was further evaluated using FITC-dextran. As shown in **Supplementary Figure S2**, DSS-induced UC model could significantly exacerbate intestinal permeability and induce a higher amount of FITC-dextran in serum compared with the control rats. However, the raised serum level of FITC in UC rats was remarkably reduced by the administration of high-dose PAE. The results were in line with the fact that PAE could regulate the expressions of tight junction-associated proteins. Besides, oral administration of PAE could restore the disturbed intestinal barrier function, by partly improving the tight junctions and up-regulating their associated proteins in the inflamed colon tissues.

Furthermore, based on the recognized relationship between IBD and intestinal immunity, more researches focusing on the regulation of intestinal microbiome associated with UC have been carried out, with the intestinal microflora being recorded as the highest intestinal immune system. Particularly, the intestinal microbiota has been demonstrated as one of the critical factors to influence nutrient metabolism and immune response, and keep the host healthy in various intestinal diseases (Zhang M. et al., 2017). The microbial diversity has been found in IBD patients and healthy individuals, with the diversity, stability and clusters in IBD patients being notably reduced. According to a previous report (Rajilic-Stojanovic et al., 2007), the gut microbiota mainly consists of *Firmicutes*, *Bacteroidetes*, *Actinobacteria*, *Proteobacteria*, and *Fusobacteria*, among which, *Bacteroidetes* and *Firmicutes* predominate and account for ∼90% of the total gut microbiota. However, based on direct gene sequencing technology by real time PCR, the proportions of these commensal bacteria could vary greatly in IBD patients. Microbial dysbiosis is generally found in UC patients, which is characterized by a reduction of bacterial diversity and an increase in the ratio of *Bacteroidetes/Firmicutes*. The intestinal bacteria, such as *Bacteroides, Eubacterium*, and *Lactobacillus*, could be reduced in IBD (Ott et al., 2004). The decreased ratio of *Firmicutes*/*Bacteroidetes* was found in TNBS-induced colitis rats (Tao et al., 2017). In our present study, based on the 16S rRNA gene sequence analysis of samples from the normal group, the DSS-induced UC group and sequential PAE treatment groups, the predominant intestinal bacteria profiles were greatly diversified. Gut microbiota community in various group at class, order and family level, respectively, were exhibited in **Supplementary Figure S3**. **Figures 7**, **8** show that the DSS-induced UC is often accompanied by shifts in gut microbiota structure, with a significant decrease of intestinal bacterial diversity, a reduction of *Firmicutes* and an increase of *Bacteroidetes* amounts, which have also been found in a previous study (Yeom et al., 2016). However, when compared with the UC rats treated with PAE, a much higher abundance of order *Lactobacillales* showed up, with the well-known probiotic properties. It has been reported that the strains of *Lactobacillus* subspecies could reduce mucosal permeability, prevent colitis onset, and alleviate inflammatory reaction (Madsen et al., 1999). The activity of PAE to increase *Lactobacillales* in UC rats would further verify its intestinal barrier improvement and anti-inflammation effects.

To evaluate the intestinal immunity regulation effect, multivariate direct gradients were used to analyze the relationship between inflammatory factors/cytokines in colonic epithelial tissue samples and intestinal microflora (**Figure 9**). As expected, protective bacteria such as *Pectobacterium*, *Roseburia*, and *Lactobacillus* were significantly negatively correlated with inflammatory cytokines such as IL-8, IL-6, TNF-α, and IFN-γ, while aggressive bacteria such as *Prevotellaceae UCG-001*, *Massilia, Bacteroides, Parabacteroides, Hymenobacter*, and *Ruminococcaceae UCG-005* were positively correlated with pro-inflammatory cytokines such as IL-1β, IL-8, IL-6, TNF-α and IFN-γ. More importantly, these evaluated inflammatory cytokines were secreted by helper T cells, also called CD^4+^ T cells, which served an important part in the immune system (Gaboriau-Routhiau et al., 2009). CD^4+^ T cells were also confirmed as an important pathogenesis of IBD, meaning that the imbalance of maintenance-regulated factor/cytokine expressions in CD^4+^ T cells might be one of the main mechanisms of disease. To be more specific, IFN-γ, IL-1β, and TNF-α were secreted by Th1, IL-6 by Th2, and IL-17 by Th17. In UC rats, both Th1/Th2 and Th17/Treg were up-regulated due to the remarkably increased effects on pro-inflammatory factors (**Figure 5**). Nevertheless, the unbalanced Th1/Th2 and Th17/Treg ratios could be reduced by PAE treatment, indicating the anti-inflammation and intestinal immunity regulation effects of PAE.

## Conclusion

In view of the recognized efficacy of *Kangfuxin* liquid, a famous commercial product made from *Periplaneta americana* extract, in the clinical treatment of gastrointestinal ulcers, the suppression effect of *P. americana* on experimental UC and the related mechanisms were investigated in the present study. Nine physiological small molecules in PAE, including cytimidine, uracil, cytidine, hypoxanthine, uridine, thymine, adenine, inosine, and guanosine, were simultaneously determined by HPLC as the quality control approach. According to the DSS-induced UC model, the administration of PAE could prominently ameliorate the intestinal damage and the associated DAI. The anti-UC mechanism of PAE could assist to inhibit inflammatory cytokines production in colon, resist oxidative stress, preserve intestinal barrier integrity and regulate the disturbed gut microbiota structure. Specifically, PAE could decrease a series of inflammatory factors such as TNF-α and IL-1β in RAW 264.7 macrophage and in colons of UC rats. The activation of Keap1/Nrf-2 pathway and the protection effect of tight junction integrity of PAE were observed. Interestingly, DSS treatment could result in significant dysbacteriosis in UC rats in comparison with normal rats, with the significantly increased *Bacteroidetes* and decreased *Firmicutes being the representative characteristics*. Nevertheless, the *Firmicutes/Bacteroidetes* ratio could be down-regulated by the administration of PAE. It could also increase the amounts of probiotics like *Lactobacillus*, which could help to modulate inflammatory bowel disorder, depress pro-inflammatory cytokine secretion, and improve intestinal barrier function. These results suggested that PAE, a complementary and alternative medicine, could be a potential pharmaceutical candidate to ameliorate UC.

## Ethics Statement

Adult male Wistar rats (180–220 g) were purchased from the Laboratory Animal Services Center, Chengdu, China. Animals in our experiments were conducted in accordance with internationally accepted guide lines for the use and care of laboratory animals, as well as those for animal experimentation issued and approved by the administrative committee of laboratory animals in Chengdu University of Traditional Chinese Medicine. Animals were housed under a standard environment condition with a temperature of 20–25°C and a 12 h dark/light cycle, and allowed free access to sterilized water and standard food.

## Author Contributions

XM and XL contributed to the animal experiments. YH and XZ contributed to the quality control by HPLC. JZ and YW contributed to data analysis and manuscript writing. CF and FG contributed to the conception and experiment design.

## Conflict of Interest Statement

The authors declare that the research was conducted in the absence of any commercial or financial relationships that could be construed as a potential conflict of interest.
