# Supplementary material for: Periplaneta americana Ameliorates Dextran Sulfate Sodium-Induced Ulcerative Colitis in Rats by Keap1/Nrf-2 Activation, Intestinal Barrier Function, and Gut Microbiota Regulation
Source: Front Pharmacol. 2018 Aug 22;9:944. doi: 10.3389/fphar.2018.00944 (PMC6113651; doi:10.3389/fphar.2018.00944)
Supplement: Supplementary file 1 [file Presentation_1.PDF]

Supplementary material

Periplaneta Americana **ameliorates dextran sulfate sodium-induced ulcerative colitis in rats by Keap1/Nrf-2 activation, intestinal barrier function and gut microbiota regulation**

Xuewei Ma[1†], Yichen Hu[2†], Xin Li[1], Xiaoting Zheng[3], Yitao Wang[4], Jinming Zhang[1,4*], Chaomei Fu[1*], Funeng Geng[5*]

1 School of Pharmacy, Chengdu University of Traditional Chinese Medicine, Chengdu 611137, China

2 College of Pharmacy and Biological Engineering, Chengdu University, Chengdu 610106, China

3 International association of quality research in Chinese medicine, Macau 999078, China

4 State Key Laboratory of Quality Research in Chinese Medicine, Institute of Chinese Medical Sciences, University of Macau, Macau 999078, China

5 Sichuan Key Laboratory of Medical American Cockroach, Chengdu 610000, China

† These authors contributed equally to the work

*** Correspondence Authors:**

Dr. Jinming Zhang. School of Pharmacy, Chengdu University of Traditional Chinese Medicine, Chengdu 611137, China.

E-mail: zhangjinming1987@126.com

Dr. Chaomei Fu. School of Pharmacy, Chengdu University of Traditional Chinese Medicine, Chengdu 611137, China.

E-mail: chaomeifu@126.com

Dr. Funeng Geng. SiWei Building B block, Yingmenkou road No.88, Chengdu 610000, Sichuan Province, China.

E-mail: haoyishenggfn@126.com

Materials and Methods

Assessment of RAW264.7 cell viability

The cytotoxicity of PAE with various concentrations was evaluated by MTT test. 5×10^5 of RAW 264.7 cells were seeded in triplicates in 96-well plates and cultured 24 h to allow cell attachment. And then, PAE at different concentrations of 0.25, 0.5, 1, 2, and 3 μg/mL were added to the wells with an additional incubation for 24 h. Subsequently, all culture medium with or without PAE was removed and 100 μL of MTT solution (1 mg/mL) was added to each well with further incubation for 4 h at 37 °C. The supernatant was discarded and 150 μL of DMSO were added to each well. Absorbance value was estimated at 570 nm by a microplate reader. Cell viability was calculated by the ratio of absorbance value of treatment group and that of blank control group.

Results

Effect of PAE on cell viability of RAW 264.7 cells

MTT test was employed to evaluate the effect of PAE on cell viability of RAW 264.7 cells. Five concentrations of PAE were incubated with RAW 264.7 cells for 24 h. As shown in Figure S1, when the concentration was lower than 2 μg/mL, PAE had no adverse effects on cell growth. However, both 2 and 3 μg/mL of PAE could obviously inhibit cell proliferation, suggesting that the concentrations of PAE at 0.25, 0.5, and 1 μg/mL would scarcely induce cell damage. The effect of PAE on the pro-inflammatory factors in LPS-stimulated RAW 264.7 cells could be investigated at these dosages.

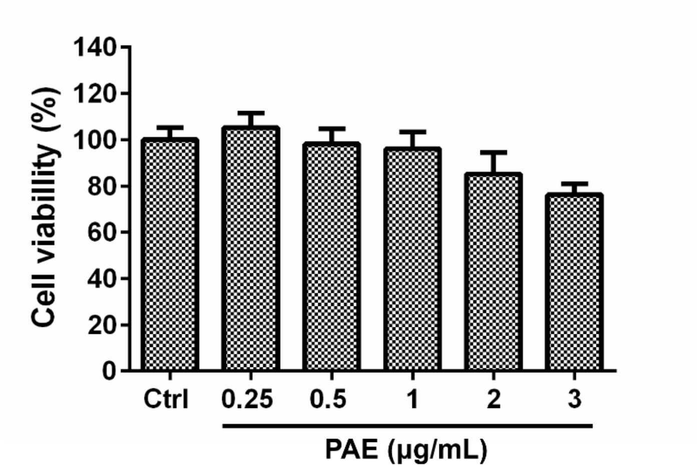

Figure S1. The effects of PAE at a series of concentrations on viability of RAW264/7 cells

for 24 h treatment.

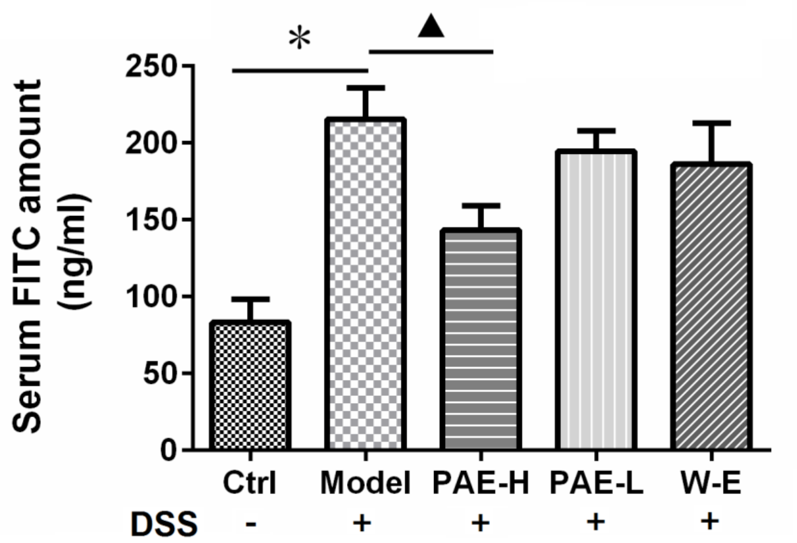

Figure S2. Amounts of FITC-dextran in the serum of DSS-induced UC mice treated with FITC-dextran and various agents as an indicator of intestinal permeability. *P<0.05 control vs. DSS model group, ▲P<0.05 model vs. PAE-H treatment group.

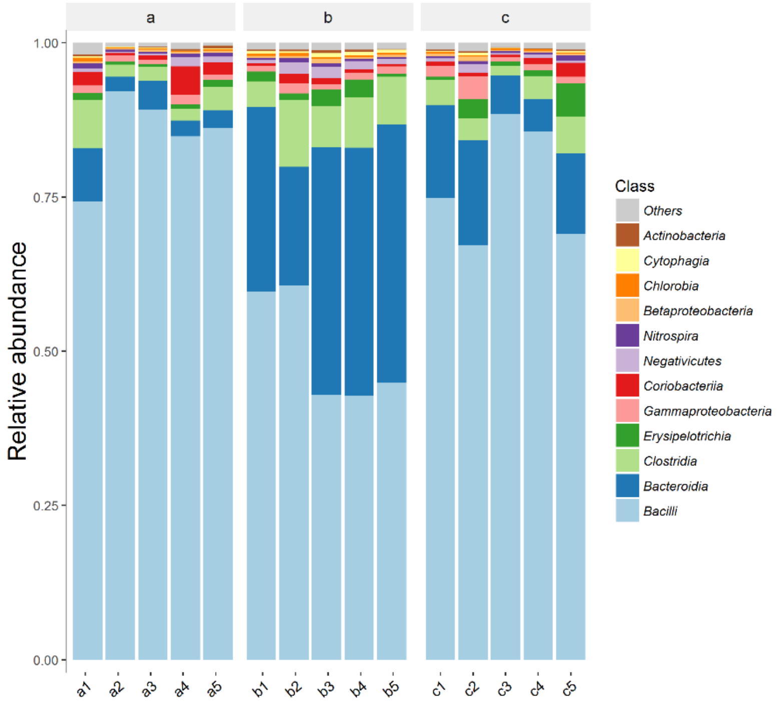

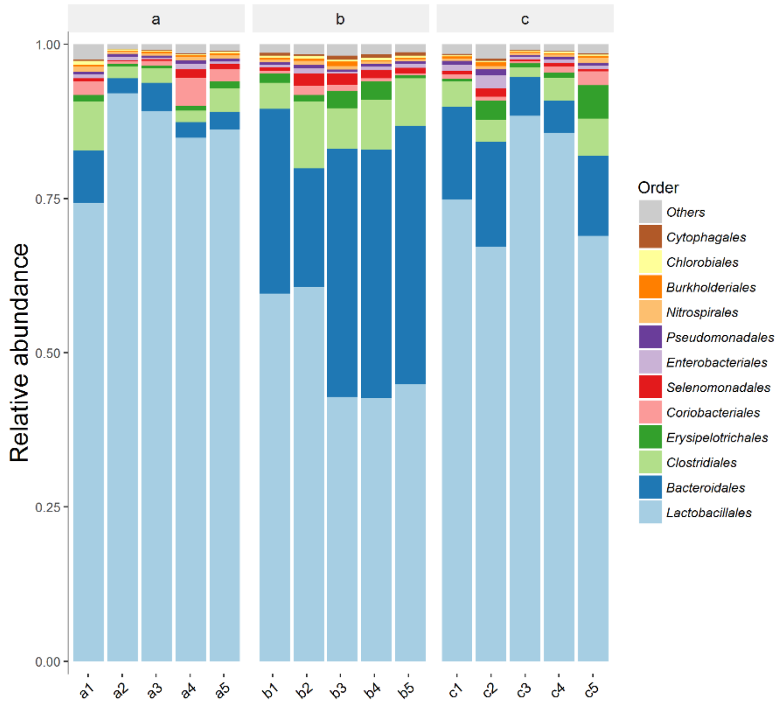

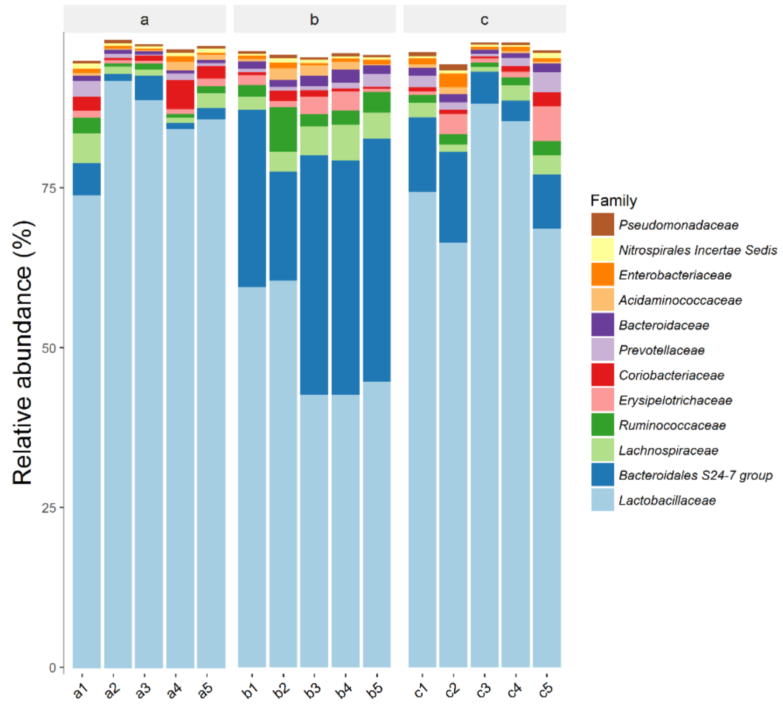

Figure S3. Gut microbiota community in normal control, DSS-induced UC model and PAE-treated group at class, order and family level, respectively.
